# ADHD medication discontinuation and non-adherence: a Norwegian population-based register study

**DOI:** 10.1136/bmjment-2026-302649

**Published:** 2026-06-22

**Authors:** Miguel Garcia-Argibay, Tore Hofstad, Ingvar Bjelland, Samuele Cortese, Arnstein Mykletun

**Affiliations:** 1 Centre for Population Health, Research Department, Division of Mental Health, Haukeland University Hospital, Bergen, Norway; 2 Developmental EPI (Evidence synthesis, Prediction, Implementation) lab, Centre for Innovation in Mental Health, Faculty of Environmental and Life Sciences, University of Southampton, Southampton, UK; 3 School of Medical Sciences, Faculty of Medicine and Health, Örebro University, Örebro, Sweden; 4 Department of Medical Epidemiology and Biostatistics, Karolinska Institutet, Stockholm, Sweden; 5 Centre for Medical Ethics, University of Oslo, Oslo, Norway; 6 Department of Clinical Medicine, University of Bergen, Bergen, Norway; 7 Hampshire and Isle of Wight NHS Foundation Trust, Southampton, UK; 8 Clinical and Experimental Sciences (CNS and Psychiatry), Faculty of Medicine, University of Southampton, Southampton, UK; 9 Hassenfeld Children’s Hospital at NYU Langone, New York University Child Study Center, New York, New York, USA; 10 DiMePRe-J-Department of Precision and Regenerative Medicine-Jonic Area, University of Bari "Aldo Moro", Bari, Italy; 11 Centre for Work and Mental Health, Nordland Hospital Trust, Bodø, Norway; 12 Department of Community Medicine, The Arctic University of Norway, Tromsø, Norway; 13 Division for Health Services, Norwegian Institute of Public Health, Oslo, Norway

**Keywords:** Neurodevelopmental Disorders, Psychopharmacology, Attention Deficit and Disruptive Behavior Disorders

## Abstract

**Background:**

Poor persistence and adherence to attention-deficit/hyperactivity disorder (ADHD) medication is a significant barrier to effective long-term care, particularly during adolescence, yet age-specific and sex-specific trajectories remain poorly characterised.

**Objective:**

To characterise medication initiation, discontinuation and long-term adherence patterns for children and adolescents diagnosed with ADHD in a real-world setting.

**Methods:**

A nationwide retrospective cohort study, including 8961 children and adolescents (aged <18 years) with a new ADHD diagnosis in child and adolescent mental health services between 1 January 2010 and 31 December 2012, with follow-up until 31 December 2021. Main outcomes were medication initiation rates; time to first medication discontinuation, analysed using Kaplan-Meier estimates and restricted mean survival time at 1 year and longitudinal adherence, measured by the proportion of days covered over 9 years.

**Findings:**

Of the 8961 individuals in the cohort (mean age at diagnosis, 12 years; 69% male), 6661 (74.3%) initiated medication, with a median time from diagnosis to initiation of 106 days (IQR 17–231); 55% initiated within 90 days. Discontinuation increased significantly with age; adolescents aged 15–17 years remained on treatment for 31.9 fewer days (95% CI −40.8 to −23.1; p<0.001) in the first year compared with children aged 5–11 years. Females also discontinued significantly earlier than males (difference −13.2 days; 95% CI −19.8 to −6.5; p<0.001). Longitudinal analysis confirmed that older age at initiation and female sex were associated with a significantly steeper decline in medication coverage over time.

**Conclusions:**

Discontinuation and low adherence to ADHD medication were common and increased substantially through adolescence, with females at higher risk of early cessation.

**Clinical implications:**

Late adolescence warrants closer clinical monitoring and shared decision-making to support appropriate treatment continuation or well-informed discontinuation, particularly for older adolescents and females. Integrating structured transition planning and attention to sex-specific barriers may help reduce avoidable non-adherence during this high-risk period.

**WHAT IS ALREADY KNOWN ON THIS TOPIC**

- While poor adherence to attention-deficit/hyperactivity disorder (ADHD) medication is a recognised barrier to effective long-term care, real-world data characterising age-specific and sex-specific longitudinal adherence trajectories remain limited.

**WHAT THIS STUDY ADDS**

- In this nationwide cohort study of 6661 Norwegian youth initiating ADHD medication, medication discontinuation was significantly higher in older adolescents and females compared with younger children and males. These high-risk groups also demonstrated a steeper decline in medication adherence over time.

**HOW THIS STUDY MIGHT AFFECT RESEARCH, PRACTICE OR POLICY**

- Older adolescents and females represent high-risk groups for discontinuing ADHD medication, highlighting clinical groups for which closer clinical monitoring and assessment of treatment benefit, functional impairment and patient preferences may help ensure discontinuation decisions are well-informed.

## Introduction

 Attention-deficit/hyperactivity disorder (ADHD) is a prevalent neurodevelopmental condition characterised by persistent patterns of inattention, hyperactivity and impulsivity that significantly impair functioning and development across various life domains.^1^ With an estimated worldwide prevalence of approximately 5–7% in children and 2.5% in adults,^1 2^ ADHD represents a considerable public health concern.

Pharmacological interventions, particularly stimulant medications, are established as a first-line treatment strategy for managing ADHD symptoms.^3^ Extensive clinical trial evidence supports the short-term efficacy of these medications in reducing core symptoms and improving functional outcomes in children, adolescents and adults.^4^ However, despite the recognised efficacy of pharmacological treatments, the translation of these short-term benefits into sustained long-term improvements is critically dependent on consistent medication use.^5^,^8^ Importantly, a growing body of evidence suggests that sustained ADHD medication use is associated with clinically meaningful reductions in adverse outcomes, including suicidal behaviours, substance misuse, criminality and transport accidents.^7 9^ These findings suggest that premature treatment cessation may carry consequences that extend beyond the re-emergence of core ADHD symptoms.

Population-based studies revealed high rates of medication discontinuation, particularly during adolescence; for instance, a recent large-scale study reported that only 47% of adolescents continue their ADHD medication 1 year after initiation and identified older age and the transition to adulthood as key predictors of early discontinuation, although sex differences were not formally tested.^10^ While some discontinuation may reflect appropriate clinical decisions, symptom improvement or successful coping without medication, population-level patterns of early and high-rate cessation raise concerns about premature discontinuation of potentially beneficial treatment.

However, detailed characterisation of age-specific and sex-specific medication trajectories remains limited, with few studies examining how these demographic factors interact to predict discontinuation and long-term adherence patterns.^10^ Therefore, this study aimed to investigate the rates and patterns of ADHD medication initiation and discontinuation using nationwide Norwegian register data. Specifically, we examined a cohort of children and adolescents aged 5–17 years at the time of their incident ADHD diagnosis within the child and adolescent mental health clinics (CAMHS) system, seeking to identify demographic, clinical and socioeconomic variables associated with medication continuation and cessation. Additionally, we examined the time from diagnosis to first medication initiation as a distinct outcome, given that delays in treatment commencement may represent a separate barrier to care that is conceptually and clinically distinct from post-initiation adherence. We hypothesised that discontinuation and non-adherence would increase with age at initiation, and that females might show differential persistence patterns given their distinct clinical presentation.^11^ Throughout this study, initiation refers to the first ADHD medication dispensing following diagnosis; discontinuation denotes a ≥90 day gap after the estimated end of medication supply; persistence refers to the duration of continuous treatment before first discontinuation and adherence describes longitudinal medication coverage measured by the proportion of days covered (PDC).

## Methods

### Study population and data sources

This study used a retrospective cohort design based on Norwegian nationwide administrative health registry data from 1 January 2009 to 31 December 2021. We identified a cohort of individuals aged 5–17 years who had contact with a CAMHS and received an incident diagnosis of ADHD (International Classification of Diseases version 10[ICD-10]: F90). The incident case definition was based on the absence of a specialist-recorded F90 diagnosis during the 1-year look-back period (2009). Additionally, individuals with ADHD medication dispensings recorded in the Norwegian Prescription Database (NorPD) during the 1-year period preceding the inclusion window were excluded, ensuring that both diagnostic and treatment history were used to confirm incident status. The inclusion period for this incident diagnosis was restricted to 1 January 2010 through 31 December 2012. This window was chosen to enable a 1-year look-back period (2009) for confirming new cases and assessing baseline medication history, while also guaranteeing a minimum of 9 years of follow-up for every individual until the end of the observation period on 31 December 2021 (the study population selection process, including the number of individuals excluded at each step, is detailed in online supplemental figure 1). Notably, all ADHD diagnoses in our cohort were recorded within specialist health services, where diagnoses follow comprehensive, multi-informant clinical assessments in accordance with national guidelines, lending high diagnostic validity to the ICD-10 codes used in this study. The longitudinal dataset was constructed by linking several national registries via each resident’s unique personal identification number. Diagnoses and specialist health contacts, including CAMHS consultations, were sourced from the Norwegian Patient Registry (NPR). Prescription data, including drug type (classified by the Anatomical Therapeutic Chemical system), dispensing date and quantity dispensed, were obtained from the NorPD, available from 1 January 2009 to 31 December 2021. Demographic details (age, sex, municipality) were retrieved from the Norwegian Population Registry, and parental education level from Statistics Norway.

### Outcomes

The primary outcomes under investigation concerned the initiation, discontinuation and overall patterns of ADHD medication use following an incident diagnosis. Medication initiation was defined as the first dispensing of any ADHD-specific medication recorded in the NorPD subsequent to the date of the incident ADHD diagnosis. We specifically evaluated initiation rates during four post-diagnosis intervals: within the first 3 months, at 4–6 months, at 7–12 months and beyond 12 months. ADHD medications included stimulants (methylphenidate, amphetamine, dexamphetamine and lisdexamphetamine) and non-stimulants (atomoxetine and guanfacine).

Among those individuals who initiated pharmacotherapy, medication discontinuation was defined as the occurrence of a gap of 90 days or more between the calculated end date of supply for a given prescription and the dispensing date of the subsequent prescription for any ADHD medication.^12 13^ Importantly, this gap represents 90 days without any estimated medication coverage, not 90 days between two consecutive prescription dates. The expected end date of supply was estimated based on the quantity of medication dispensed, assuming a standard daily dose of one defined daily dose. Switches between different ADHD medications (eg, between stimulants or from a stimulant to a non-stimulant) were treated as treatment continuation, provided the gap between the estimated end of supply of the preceding medication and the dispensing of the new medication was less than 90 days. The primary measure for this outcome was the time from the initial medication dispensing to the first such discontinuation event, observed over a follow-up period extending until 31 December 2021. Individuals were censored at death, emigration or end of follow-up period, whichever happened first.

### Covariates

A range of covariates for medication initiation and discontinuation were extracted from the linked registry data to explore factors associated with these outcomes. Demographic factors considered included the individual’s age at the time of the incident ADHD diagnosis, which was categorised into relevant developmental stages (5–11 years, 12–14 years, 15–17 years) and sex. Socioeconomic status was represented by the highest attained parental education level (categorised as low (up to lower secondary), medium (upper secondary/vocational) and high (tertiary)).

Clinical factors included the presence of co-occurring psychiatric disorders, identified through any recorded ICD-10 codes within the F-chapter (Mental and Behavioral Disorders) in the NPR during the 12 months preceding the initiation of ADHD pharmacotherapy, excluding the primary F90 ADHD diagnosis. These codes were summed for each participant and classified into three categories: no psychiatric comorbidity and one or multiple psychiatric comorbidities (≥2). For analyses focusing specifically on medication discontinuation, additional medication-related factors were examined, notably the type of ADHD medication first initiated (stimulant vs non-stimulant). In all stratified analyses by medication class, the classification refers to the first ADHD medication initiated following diagnosis.

### Statistical analysis

Baseline characteristics were summarised using counts and percentages for categorical variables and medians with IQRs for continuous variables, overall and stratified by medication initiation status, with χ^2^ tests for bivariate associations. To provide further clinical context, we examined the temporal distribution of medication initiation relative to diagnosis date, categorising the lag time between diagnosis and first prescription as short-term (within 3 months), medium-term (4–6 months), long-term (7–12 months) and delayed (>12 months). We also characterised the initial prescribing patterns, including specific medication and class across different age strata to establish the treatment landscape at baseline. Finally, we summarised discontinuation trajectories by grouping patients into four mutually exclusive categories (ie, discontinuation within 1 year, discontinuation between 1 year and 2 years, discontinuation ≥ 3 years and continued treatment) and reported the number of individuals and corresponding percentages in each group.

The cumulative incidence of the first discontinuation event was estimated using the Kaplan-Meier method. Stratified analyses were conducted across demographic and clinical subgroups to identify potential heterogeneity in discontinuation patterns. Specifically, we stratified by age at initiation (5–11 years, 12–14 years and 15–17 years), sex, medication class at initiation (stimulant vs non-stimulant), presence of psychiatric comorbidities and parental socioeconomic status (using education level as proxy). The log-rank test was used to assess differences in Kaplan-Meier curves between strata.

To assess differences in medication continuation over time across patient subgroups, restricted mean survival time (RMST) analysis was performed at 1 year of follow-up. This timeframe was selected as it represents a critical period for establishing treatment effectiveness and tolerability and it provides a clear, quantifiable measure of early discontinuation that complements the long-term view of the Kaplan-Meier curves. The RMST represents the average survival time from baseline up to a specified time horizon and is calculated as the area under the Kaplan-Meier survival curve up to that time point. This approach provides a clinically interpretable measure of absolute treatment effect that does not require the proportional hazards assumption.^14^ We performed pairwise comparisons for key stratification variables. The reference group for each comparison was defined as: 5–11 years (vs 12–14 and 15–17 years), male (vs female), stimulant medication (vs non-stimulant), no psychiatric comorbidities (vs one and multiple) and low parental education (vs medium and high). RMST estimates with 95% CIs were calculated for each group. Bootstrap resampling with 1000 iterations was employed to estimate standard errors and construct CIs for the RMST values.

To capture the dynamic nature of ADHD medication use (where periods of discontinuation and reinitiation are common),^10 15^ we complemented the survival analysis with longitudinal adherence measurement using the PDC.^16^ To prevent double-counting from overlapping fills and thus, PDC inflation, each prescription’s coverage start date was adjusted to the maximum of either its dispensing date or the day after the previous fill ended. The observation period was divided into sequential 90-day intervals. For each individual and interval, PDC was calculated as the total adjusted coverage days divided by 90, capped at 1.0. Adherence trajectories were visualised using mean PDC line plots across intervals, stratified by the aforementioned variables. To test differences in PDC over time, we fit a linear mixed-effects model with a random intercept for each participant, allowing us to account for within-person correlation while estimating temporal trends and covariate effects. All statistical analyses were conducted using R V.4.5.0.^17^ An overview of all medication-related analyses, including their objectives, outcome measures and time horizons, is provided in online supplemental table 1.

## Results

### Cohort characteristics and medication initiation

The study cohort included 8961 children and adolescents diagnosed with ADHD in Norwegian CAMHS between January 2010 and December 2012. The mean age at ADHD diagnosis was 12 years (IQR: 8.8–14.6), and 69% (n=6177) of the cohort were male. Among these, 6661 (74.3%) initiated ADHD treatment following diagnosis. The median time from diagnosis to medication initiation was 106 days (IQR: 17–231). Most patients (55%) initiated treatment within 90 days of diagnosis, with 12% experiencing delayed initiation, commencing medication more than 1 year after receiving an ADHD diagnosis. At initiation, stimulants were the predominant medication class (98%), with non-stimulants comprising 2.4% of first prescriptions. Demographic and clinical characteristics are presented in table 1.

**Table 1:** Baseline demographic, clinical and treatment characteristics of children and adolescents with an incident ADHD diagnosis (N=8961), stratified by medication initiation status

Characteristic	Started ADHD pharmacotherapy	
	No	Yes
	N=2300 (25.67%)	N=6661 (74.33%)
Sex		
Male	1687 (73%)	4490 (67%)
Female	613 (27%)	2171 (33%)
Region of origin		
Europe	2047 (89%)	6044 (91%)
Other	253 (11%)	617 (9.3%)
Parental education		
Low (up to lower secondary)	258 (12%)	591 (9.3%)
Medium (upper secondary/vocational)	1612 (73%)	4525 (71%)
High (tertiary)	328 (15%)	1228 (19%)
Unknown	102	317
Age at ADHD diagnosis (years)	13.3 (10.7, 15.6)	10.7 (8.6, 13.8)
Age at ADHD diagnosis, years		
5–11	863 (38%)	4108 (62%)
12–14	786 (34%)	1448 (22%)
15–17	651 (28%)	1105 (17%)
Age at ADHD treatment initiation, years		
5–11	–	3764 (57%)
12–14	–	1516 (23%)
15–17	–	1209 (18%)
≥18	–	172 (2.6%)
Type of medication		
Non-stimulants	–	162 (2.4%)
Stimulants	–	6499 (98%)
Medication		
Amphetamine	–	<5 (<0.1%)
Dexamphetamine	–	16 (0.2%)
Methylphenidate	–	6471 (97%)
Atomoxetine	–	162 (2.4%)
Lisdexamphetamine	–	11 (0.2%)
Time to start of pharmacological treatment, days	–	106 (17, 231)
Time to treatment		
Within 3 months	–	3662 (55%)
4–6 months	–	1063 (16%)
7–12 months	–	1169 (18%)
>12 months	–	767 (12%)
Time to pharmacological treatment discontinuation, years	–	0.93 (0.29, 2.61)
Time to treatment discontinuation, years		
Within 1 year	–	2433 (37%)
1–2 years	–	1251 (19%)
≥3 years	–	1043 (16%
Continued	–	1934 (29%)

ADHD, attention-deficit/hyperactivity disorder.

**Figure 1:**
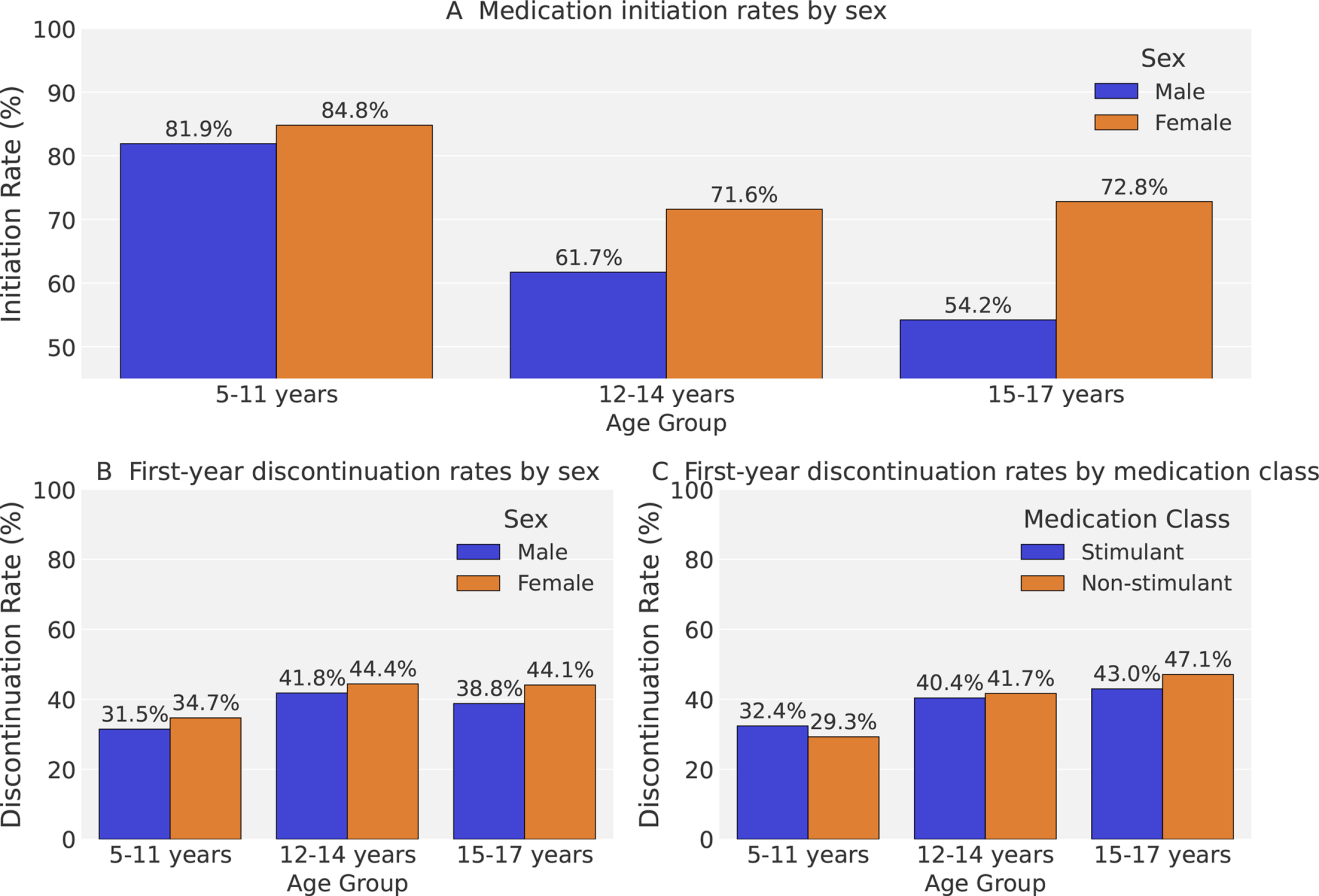
ADHD medication initiation rates by age group and sex (**A**), and first-year discontinuation rates by age group and sex (**B**) and by age group and medication class (**C**). ADHD, attention-deficit/hyperactivity disorder.

Medication initiation rates varied significantly by age and sex (figure 1A). Initiation rates were the highest in the youngest age group and decreased with age. Within each age stratum, females showed higher rates of treatment initiation than males. In the 5–11 years group, 84.8% of females and 81.9% of males initiated medication. This pattern continued for those aged 12–14 years (71.6% female vs 61.5% male) and 15–17 years (72.8% female vs 54.2% male). First-year discontinuation rates increased with age (figure 1B). Notably, in the 5–11 years age group, stimulants were associated with a slightly higher first-year discontinuation rate (32.4%) compared with non-stimulants (29.3%). In older age groups, non-stimulants had higher discontinuation rates, with rates for the 12–14 years group at 40.4% for stimulants and 41.7% for non-stimulants, and for the 15–17 years group at 43.0% for stimulants and 47.1% for non-stimulants.

Kaplan-Meier survival analyses were performed to estimate the cumulative incidence of medication discontinuation over a 10-year follow-up period (online supplemental figure 2). A significant positive association was found between age at treatment initiation and time to discontinuation (log-rank p<0.001). Adolescents aged 15–17 years had the highest cumulative incidence of discontinuation, followed by those aged 12–14 years, while children aged 5–11 years demonstrated the highest persistence. A significant difference in discontinuation was also observed between sexes (log-rank p<0.001), with females showing a slightly higher cumulative incidence of discontinuation over time compared with males. No statistically significant associations were found for medication class (p=0.52), number of psychiatric comorbidities (p=0.37) or parental education level (p=0.26).

RMST analysis at 1 year quantified clinically meaningful differences in treatment persistence (table 2). Compared with children aged 5–11 years (RMST: 290.3 days), adolescents had significantly shorter treatment durations. Those aged 12–14 years received an average of 17.6 fewer days of medication (95% CI −25.3 to −9.9), while older adolescents aged 15–17 years received 31.9 fewer days (95% CI −40.8 to −23.1). A significant difference was also found for sex, with females remaining on treatment for an average of 13.2 fewer days than males (95% CI −19.8 to −6.5). No statistically significant differences in mean time on medication at 1 year were observed for medication class, number of psychiatric comorbidities or parental education level.

**Table 2:** Restricted mean survival time (RMST) analysis at 1 year: pairwise comparisons of time to first ADHD medication discontinuation across demographic, clinical and socioeconomic subgroups (N=6661)

Variable	Comparison	N ref	N comp	RMST ref (95% CI)	RMST comp (95% CI)	Difference (95% CI)
Age group, years	12–14 versus 5–11	3764	1516	290.29 (286.34 to 294.05)	272.71 (265.97 to 279.10)	−17.58 (−25.31 to −9.85)*
	15–17 versus 5–11	3764	1209	290.29 (286.42 to 293.98)	258.36 (249.92 to 265.75)	−31.94 (−40.75 to −23.13)*
Sex	Female versus male	4490	2171	283.84 (280.06 to 287.59)	270.67 (265.06 to 275.66)	−13.17 (−19.82 to −6.52)*
Medication class	Non-stimulants versus stimulants	6499	162	279.96 (277.20 to 282.89)	263.26 (240.32 to 284.07)	−16.70 (−39.26 to 5.87)
Number of psychiatric comorbidities	One versus none	6049	575	279.05 (276.04 to 282.38)	284.47 (274.61 to 294.99)	5.42 (−5.19 to 16.03)
	Multiple versus none	6049	37	279.05 (275.95 to 282.08)	284.59 (241.86 to 322.54)	5.54 (−35.59 to 46.68)
Parental education	Medium versus low	591	4525	274.30 (264.40 to 285.39)	280.12 (276.51 to 283.68)	5.82 (−5.28 to 16.91)
	High versus low	591	1228	274.30 (265.55 to 285.02)	279.89 (272.38 to 287.10)	5.59 (−6.81 to 17.98)

* Displays statistical significant differences. ref=reference group; comp=comparison group.

ADHD, attention-deficit/hyperactivity disorder; comp, comparison group; ref, reference group.

Linear mixed-effects models showed significant heterogeneity in adherence trajectories across demographic subgroups (online supplemental figure 3). After accounting for baseline adherence, older age at initiation was associated with significantly steeper declines in PDC over time. For instance, within 3 years, compared with children aged 5–11 years, those aged 12–14 years experienced an additional 2.5 percentage point decrease per 90-day interval (95% CI −0.027 to −0.023; p<0.001), while adolescents aged 15–17 years showed an even greater decline of 4.1 percentage points per interval (95% CI −0.043 to −0.039; p<0.001). Similarly, females exhibited a slightly steeper rate of decline compared with males (difference: −0.6 percentage points per interval; 95% CI −0.008 to −0.005; p<0.001). In contrast, higher parental education was associated with attenuated decline in adherence. Both medium and high parental education levels demonstrated significantly slower rates of PDC decrease compared with low education (0.7 and 0.6 percentage points per interval, respectively; both p<0.001). Neither medication class at initiation nor the presence of psychiatric comorbidities was significantly associated with the rate of change in adherence over the observation period (both p>0.4; see table 3 for comparisons at the end of follow-up).

**Table 3:** Results of the linear mixed-effects model for the rate of change in proportion of days covered (PDC)

Subgroup	Comparison	3 years		9 years	
		PDC difference	P value	PDC difference	P value
Sex	Female versus male	−0.006 (−0.008 to −0.005)	0.000	0.000 (−0.001 to 0.000)	0.061
Medication class	Non-stimulants versus stimulants	0.003 (−0.002 to 0.008)	0.273	−0.004 (−0.005 to −0.003)	0.000
Age	12-14 versus 5–11	−0.025 (−0.027 to −0.023)	0.000	−0.012 (−0.012 to −0.011)	0.000
Age	15-17 versus 5–11	−0.041 (−0.043 to −0.039)	0.000	−0.008 (−0.009 to −0.008)	0.000
Parental education	High versus low	0.006 (0.003 to 0.009)	0.000	0.002 (0.001 to 0.003)	0.000
Parental education	Medium versus low	0.007 (0.004 to 0.010)	0.000	0.002 (0.002 to 0.003)	0.000
Number of psychiatric comorbidities	Multiple versus none	−0.006 (−0.016 to 0.004)	0.229	−0.008 (−0.01 to −0.006)	0.000
Number of psychiatric comorbidities	One versus none	0.001 (−0.002 to 0.004)	0.493	0.000 (0.000 to 0.001)	0.830

## Discussion

In this population-based cohort study of Norwegian children and adolescents diagnosed with ADHD, we found that while most individuals (74%) initiated pharmacological treatment following their diagnosis, this initiation was often delayed. Just over half (55%) began treatment within 3 months, yet the median time to the first prescription was 106 days, and a substantial minority (12%) waited over a year. For those who did start treatment, we found that continuation over time was a subsequent major challenge, with discontinuation being common and increasing markedly with age. Adolescents, particularly those aged 15–17 years, had the highest rates of discontinuation. The RMST analysis suggested that these differences to be clinically significant: older adolescents discontinued treatment a full month earlier than younger children within the first year, and females discontinued nearly 2 weeks earlier than males. The RMST sex difference (~13 days) is modest compared with the age effect (~32 days), but its convergence across all three analytical approaches and the progressive widening of the adherence gap in the PDC analysis supports its clinical relevance. Notably, while a recent multinational study presented sex-stratified survival curves as shown in online supplemental material, no formal statistical testing of sex differences was performed, making our quantification of sex-specific trajectories a novel contribution. Furthermore, our longitudinal adherence analysis, which tracked medication coverage over 9 years, confirmed that this disengagement intensified over time; older age and female sex were associated with a significantly steeper decline in adherence. The observed initiation pattern also warrants consideration. While most individuals commenced treatment within 3 months, the median time was 106 days and 12% waited over a year. Delayed initiation represents a conceptually distinct barrier from poor post-initiation adherence, requiring different clinical interventions and meriting dedicated investigation. Parental education and psychiatric comorbidity were not significantly associated with discontinuation in the survival or RMST analyses, although the PDC analysis revealed modestly slower adherence decline with higher parental education.

An important finding is the pattern observed in females, who demonstrated higher rates of medication initiation across all age groups but also discontinued treatment significantly earlier than males within the first year. This may reflect several underlying factors. Females with ADHD are more likely to present with internalising symptoms and co-occurring anxiety or depression, which might prompt initial treatment but also complicate long-term management.^11^ However, broadly defined psychiatric comorbidity was not significantly associated with adherence decline in our analyses, suggesting that earlier female discontinuation may operate through pathways not captured by this measure, such as differential side-effect profiles, perceived efficacy or gendered attitudes toward long-term medication use. This finding challenges a simplistic view of treatment access and suggests that simply starting medication is not a sufficient metric of care quality for females with ADHD. This is further highlighted by our adherence trajectory analysis, which revealed that females also exhibit a steeper decline in medication coverage over time, suggesting that the factors driving their disengagement are persistent and cumulative.

Our findings align with and extend the results of previous large-scale research. A recent multinational study similarly reported that early medication discontinuation is prevalent, with persistence being the lowest among adolescents and young adults.^10^ Their finding of a peak in discontinuation around ages 18–19 years represents a natural continuation of the trend we observed in our 15–17 year-old group, highlighting the transition to adulthood as a period of vulnerability for treatment cessation. This is likely a multifactorial phenomenon. Adolescence is a developmental stage characterised by a desire for autonomy, heightened sensitivity to social stigma and the formation of self-identity, all of which can lead to questioning the need for medication. For older adolescents, taking daily ADHD medication may conflict with their emerging self-concept, particularly if they perceive the medication as incompatible with the identity they wish to project or if it serves as an unwanted reminder of a diagnostic label.^18^ Our PDC analysis reinforces this point, showing not just that adolescents stop sooner but that their adherence actively deteriorates at a significantly faster rate (a decline of over 4 percentage points per 90-day period for 15–17 year-olds) compared with younger children.

The high discontinuation rates observed, especially among adolescents, raise critical questions about the reasons for cessation and its potential consequences. While some may discontinue due to symptom remission or a shared decision with their clinician, literature suggests that many cease treatment due to adverse effects, perceived lack of efficacy, stigma or practical challenges associated with the transition from paediatric to adult healthcare systems.^1519^,^21^ The question of whether, when and for whom discontinuation is harmful is vital. A growing body of evidence demonstrates that ADHD medication is associated with reduced risks of critical adverse outcomes, including suicidal behaviours, substance misuse and criminality.^7 9^ Viewed through this lens, the premature discontinuation of effective treatment is not a neutral event but a significant clinical and public health concern that may leave individuals at higher risk for negative life trajectories. Meta-analytic evidence suggests that tolerability concerns and adverse effects may increasingly outweigh perceived benefits as patients age, contributing to the high discontinuation rates observed in real-world settings.^22^

Our findings have important clinical implications and point toward clear future research directions. The sharp increase in discontinuation during adolescence highlights the potential value for closer clinical attention. A crucial next step would be the development of clinical prediction models using large-scale data to identify which patients are most likely to discontinue early. By pinpointing the most significant predictors (eg, age, medication type and comorbidity burden) these models could enable clinicians to identify individuals more likely to discontinue treatment early and to consider offering additional support where appropriate. Complementing this quantitative approach, future research should employ qualitative and mixed-methods designs to understand the patient-level and family-level drivers behind these decisions. Furthermore, studies with access to detailed clinical data (eg, ADHD symptom severity, intellectual functioning and adaptive impairment) would be particularly valuable for identifying clinical phenotypes associated with long-term non-adherence, which cannot be captured through administrative registries alone. Finally, insights from both predictive and qualitative studies could inform the development and evaluation of novel support models, such as tailored transition clinics or personalised digital health tools, aimed at supporting appropriate treatment continuity and timely, well-informed discontinuation decisions for adolescents and young adults.

The primary strength of our study is its population-based design, which uses comprehensive and highly reliable data from Norwegian national registries. This approach minimises selection bias and ensures high generalisability to a real-world clinical population, capturing all individuals diagnosed and treated within the national CAMHS. The use of prescription fill data from a national database provides an objective measure of treatment collection, avoiding the recall and reporting biases inherent in self-report studies. Furthermore, our tripartite analytical approach, combining Kaplan-Meier curves, RMST and longitudinal PDC modelling, provides a robust and multifaceted view of discontinuation and adherence patterns.

Some limitations must be considered when interpreting these results. First, our data reflect the filling of prescriptions, not the actual consumption of medication. Adherence to collecting medication is a proxy for but not a direct measure of adherence to ingesting it. While our PDC analysis provides a more nuanced view of long-term medication coverage, it still cannot confirm consumption. Second, the 1-year look-back period used to define incident ADHD diagnoses may not fully exclude all prevalent cases, particularly among older adolescents with longer disease histories. However, extending the look-back period was not feasible given the available data structure, and our approach is consistent with washout periods used in comparable pharmacoepidemiological studies.^7 10^ Importantly, ADHD diagnoses recorded in the NPR are made within specialist services, making it unlikely that a previously diagnosed individual would have no specialist registry contact for an entire year. Third, the survival analysis treated first discontinuation as a terminal event without accounting for reinitiation, possibly overestimating permanent cessation. Many individuals with ADHD engage in episodic treatment,^10^ and the longitudinal PDC analysis was designed to complement this by capturing medication coverage regardless of stop-start patterns. The absence of a significant medication class effect should be interpreted cautiously, as only 2.4% initiated non-stimulants, likely representing a clinically selected subgroup with potential confounding by indication, and the comparison was underpowered. Fourth, the data pertain to a cohort diagnosed between 2010 and 2012. Since that time, there has been a significant increase in the rates of ADHD diagnosis and medication initiation in Norway and globally.^23^ This secular trend may mean that individuals diagnosed today represent a broader spectrum of severity, potentially altering discontinuation patterns. It is plausible that with a less severely affected patient population, discontinuation rates may now be even higher. Finally, as an observational study, we lack granular clinical data, such as ADHD symptom severity, reasons for discontinuation or psychosocial factors that could influence treatment persistence. Relatedly, our data do not allow us to determine whether individuals received concurrent psychotherapy, which may independently affect medication adherence and discontinuation decisions. The NPR records healthcare contacts but does not include sufficiently detailed procedure codes to reliably distinguish structured psychotherapy from routine clinical follow-up. Finally, psychiatric comorbidity was operationalised as a count of any ICD-10 F-chapter diagnoses, collapsing clinically heterogeneous conditions into a single variable that may obscure differential associations with medication persistence.

## Conclusions

In conclusion, this nationwide study demonstrates that while ADHD medication is widely initiated, discontinuation and poor adherence are common, particularly during adolescence and in females. These findings highlight the need for closer clinical attention during adolescence and prospective research examining whether discontinuation reflects optimal treatment adjustments or missed opportunities for beneficial care.

## Supplementary material

10.1136/bmjment-2026-302649 online supplemental file 1

## Data Availability

Data may be obtained from a third party and are not publicly available.

## References

[1] Faraone SV, Banaschewski T, Coghill D (2021). The World Federation of ADHD International Consensus Statement: 208 Evidence-based conclusions about the disorder. Neurosci Biobehav Rev.

[2] Chaulagain A, Lyhmann I, Halmøy A (2023). A systematic meta-review of systematic reviews on attention deficit hyperactivity disorder. Eur Psychiatry.

[3] Cortese S (2020). Pharmacologic Treatment of Attention Deficit–Hyperactivity Disorder. N Engl J Med.

[4] Cortese S, Adamo N, Del Giovane C (2018). Comparative efficacy and tolerability of medications for attention-deficit hyperactivity disorder in children, adolescents, and adults: a systematic review and network meta-analysis. Lancet Psychiatry.

[5] Matthijssen A-FM, Dietrich A, Bierens M (2019). Continued Benefits of Methylphenidate in ADHD After 2 Years in Clinical Practice: A Randomized Placebo-Controlled Discontinuation Study. Am J Psychiatry.

[6] Buitelaar JK, Trott G-E, Hofecker M (2012). Long-term efficacy and safety outcomes with OROS-MPH in adults with ADHD. Int J Neuropsychopharmacol.

[7] Zhang L, Zhu N, Sjölander A (2025). ADHD drug treatment and risk of suicidal behaviours, substance misuse, accidental injuries, transport accidents, and criminality: emulation of target trials. BMJ.

[8] Fredriksen M, Halmøy A, Faraone SV (2013). Long-term efficacy and safety of treatment with stimulants and atomoxetine in adult ADHD: a review of controlled and naturalistic studies. Eur Neuropsychopharmacol.

[9] Widding-Havneraas T, Zachrisson HD, Markussen S (2024). Effect of Pharmacological Treatment of Attention-Deficit/Hyperactivity Disorder on Criminality. J Am Acad Child Adolesc Psychiatry.

[10] Brikell I, Yao H, Li L (2024). ADHD medication discontinuation and persistence across the lifespan: a retrospective observational study using population-based databases. Lancet Psychiatry.

[11] Quinn PO, Madhoo M (2014). A review of attention-deficit/hyperactivity disorder in women and girls: uncovering this hidden diagnosis. Prim Care Companion CNS Disord.

[12] Rasmussen L, Wettermark B, Steinke D (2022). Core concepts in pharmacoepidemiology: Measures of drug utilization based on individual-level drug dispensing data. Pharmacoepidemiol Drug Saf.

[13] Garcia-Argibay M, Chang Z, Brikell I (2025). Evaluating ADHD medication trial representativeness: a Swedish population-based study comparing hypothetically trial-eligible and trial-ineligible individuals. Lancet Psychiatry.

[14] Han K, Jung I (2022). Restricted Mean Survival Time for Survival Analysis: A Quick Guide for Clinical Researchers. Korean J Radiol.

[15] Gajria K, Lu M, Sikirica V (2014). Adherence, persistence, and medication discontinuation in patients with attention-deficit/hyperactivity disorder - a systematic literature review. *Neuropsychiatr Dis Treat*.

[16] Prieto-Merino D, Mulick A, Armstrong C (2021). Estimating proportion of days covered (PDC) using real-world online medicine suppliers’ datasets. J Pharm Policy Pract.

[17] R Core Team (2023). R: a language and environment for statistical computing. https://www.R-project.org/.

[18] Eccleston L, Williams J, Knowles S (2019). Adolescent experiences of living with a diagnosis of ADHD: a systematic review and thematic synthesis. Emot Behav Difficulties.

[19] Khan MU, Aslani P (2021). Exploring factors influencing initiation, implementation and discontinuation of medications in adults with ADHD. Health Expect.

[20] Brinkman WB, Simon JO, Epstein JN (2018). Reasons Why Children and Adolescents With Attention-Deficit/Hyperactivity Disorder Stop and Restart Taking Medicine. Acad Pediatr.

[21] Titheradge D, Godfrey J, Eke H (2022). Why young people stop taking their attention deficit hyperactivity disorder medication: A thematic analysis of interviews with young people. Child Care Health Dev.

[22] Castells X, Cunill R, Capellà D (2013). Treatment discontinuation with methylphenidate in adults with attention deficit hyperactivity disorder: a meta-analysis of randomized clinical trials. Eur J Clin Pharmacol.

[23] Hartz I, Madsstuen NHH, Andersen PN (2024). Nationwide trends in the use of ADHD medications in the period 2006-2022: a study from the Norwegian prescription database. BMC Psychiatry.

