# Supplementary material for: ADHD medication discontinuation and non-adherence: a Norwegian population-based register study
Source: BMJ Ment Health. 2026 Jun 22;29(1):e302649. doi: 10.1136/bmjment-2026-302649 (PMC13289004; doi:10.1136/bmjment-2026-302649)
Supplement: online supplemental file 1 [file bmjment-29-1-s001.docx]

**Supplementary material for: ADHD medication discontinuation and non-adherence: A Norwegian population-based register study**

Miguel Garcia-Argibay, Ph.D.^1-4*^, Tore Hofstad, Ph.D.^1,5^, Ingvar Bjelland, Ph.D.^1,6^, Samuele Cortese, MD, Ph.D.^2,7-10^, Arnstein Mykletun, Ph.D.^1,11-13^

Contents

[Supplementary Tables 2](#_Toc226639946)

[Supplementary figures 3](#_Toc226639947)

# Supplementary Tables

**Supplementary Table 1.** Summary of statistical analyses for medication initiation and adherence

| **Analysis** | **Objective** | **Outcome measure** | **Time horizon** | **Covariates** |
| --- | --- | --- | --- | --- |
| Medication initiation rates | Characterize treatment uptake following diagnosis | Initiation (yes/no) and time from diagnosis to first prescription | Full follow-up | Age, sex |
| First-year discontinuation rates | Describe early treatment cessation by subgroup | Discontinuation within 1 year (yes/no) | 1 year | Age, sex, medication class |
| Kaplan-Meier survival analysis | Estimate cumulative incidence of first discontinuation | Time to first discontinuation (≥90-day gap) | Up to 10 years | Age, sex, medication class, psychiatric comorbidities, parental education |
| RMST analysis | Quantify absolute differences in treatment duration | Restricted mean survival time at 1 year | 1 year | Same as above |
| Linear mixed-effects model | Model longitudinal adherence trajectories | Proportion of Days Covered (90-day intervals) | 9 years | Same as above |

# Supplementary figures

**Supplementary Figure 1.** Study population selection flowchart.


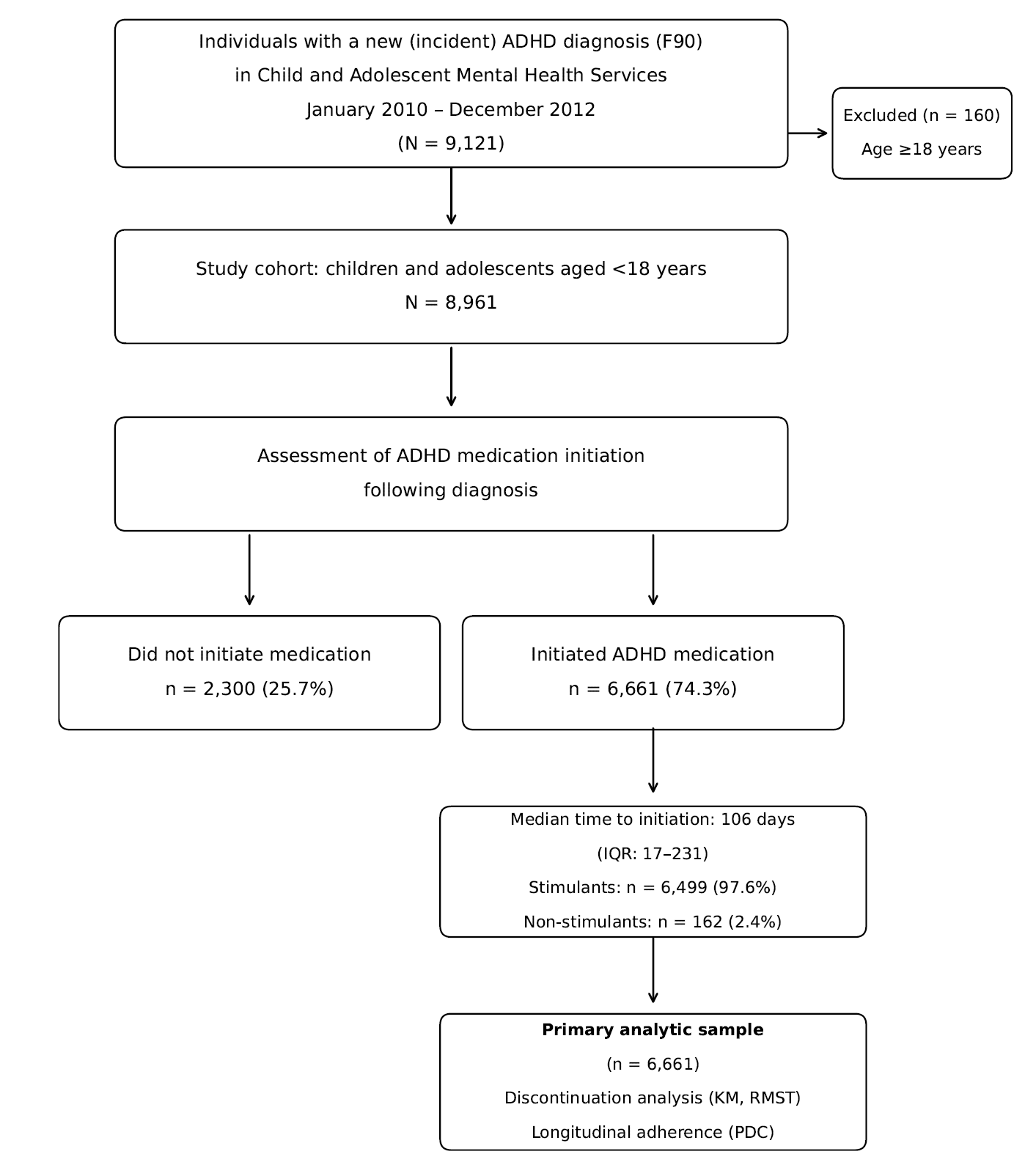


**Supplementary Figure 2.** Cumulative incidence of first ADHD medication discontinuation stratified by age group, sex, medication class, and number of psychiatric comorbidities.


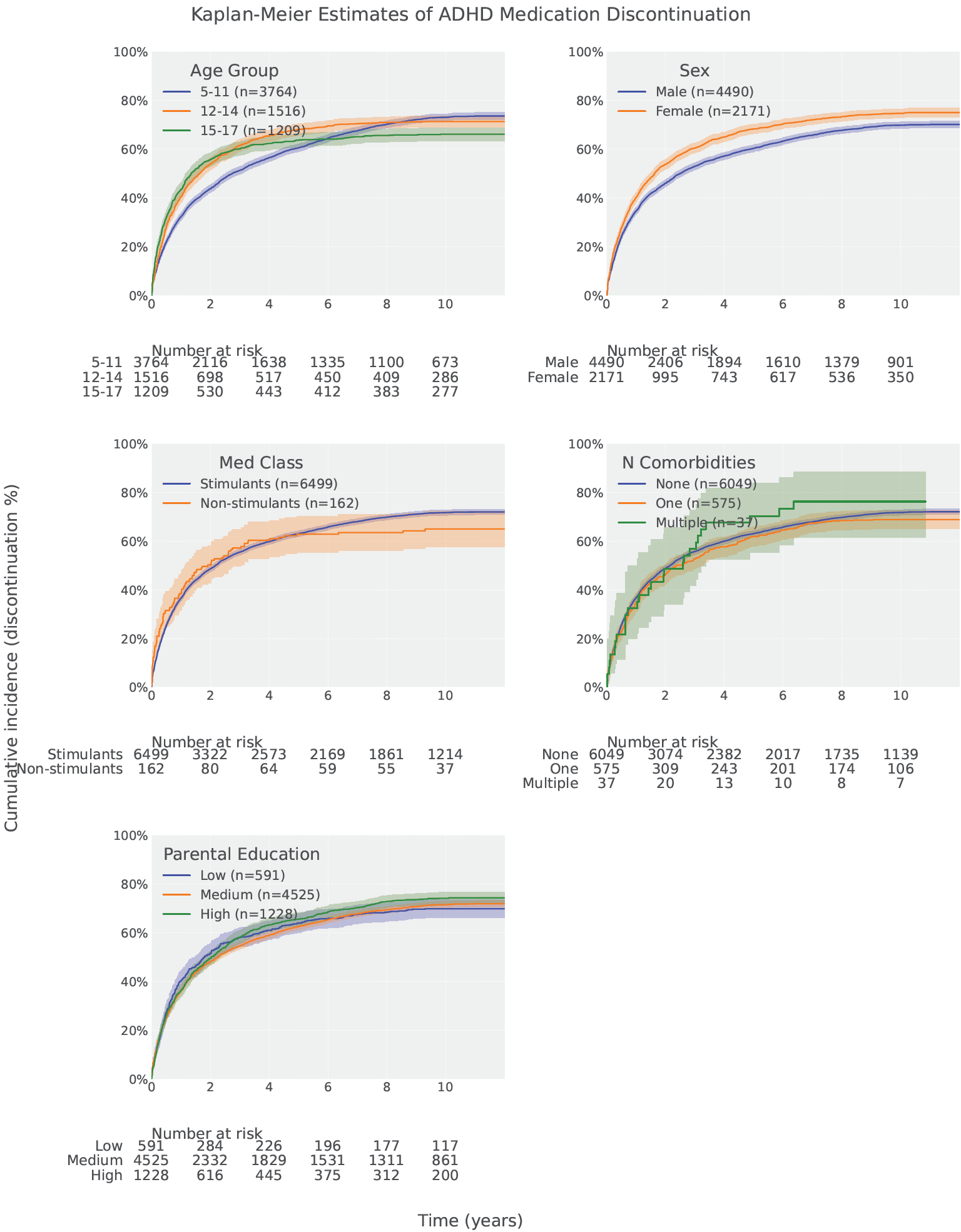


*Note.* P_logrank_ 0.064, <0.001, 0.52, 0.37, 0.26, respectively. N comorbidities refer to the number of psychiatric comorbidities.

**Supplementary Figure 3.** Mean adherence (proportion of days covered [PDC]) trajectories over a 9-year period divided in 90-day intervals stratified by age at treatment initiation, sex, medication class, number of psychiatric comorbidities, and parental education.


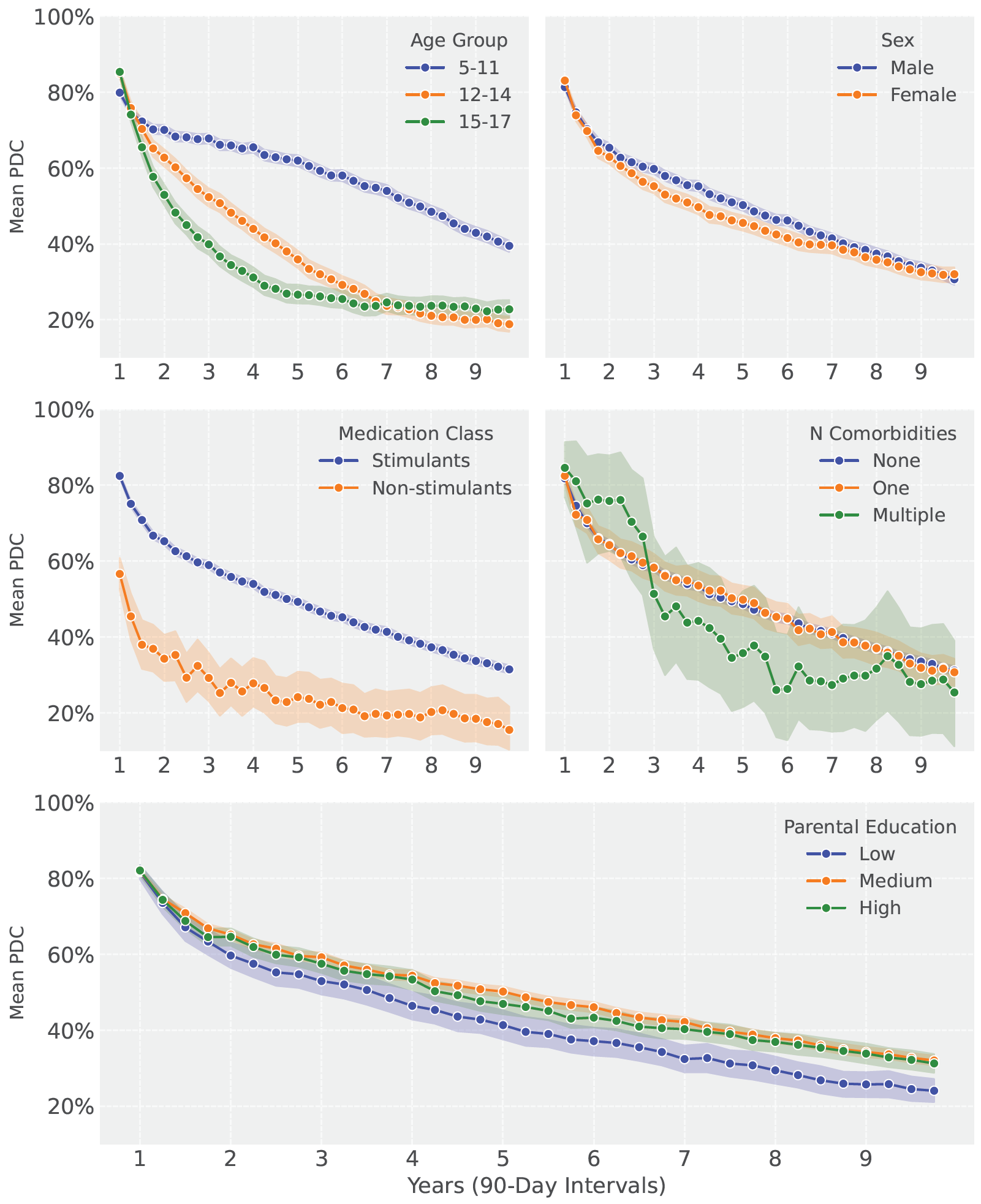


*Note.* N comorbidities refer to the number of psychiatric comorbidities.
